# Consumption and tax gains attributable to Covid-19 vaccinations in 12 EU countries with low vaccination rates

**DOI:** 10.1093/eurpub/ckad023

**Published:** 2023-02-13

**Authors:** Jonathan Cylus, Jessica Walters, Martin McKee, Peter Cowley

**Affiliations:** European Observatory on Health Systems and Policies, London, UK; LSE Health, Department of Health Policy, London School of Economics and Political Science, London, UK; Department of Health Services Research and Policy, London School of Hygiene and Tropical Medicine, London, UK; WHO Barcelona Office for Health Systems Financing, World Health Organization Regional Office for Europe, Barcelona, Spain; Barcelona Institute for Global Health (ISGlobal), Barcelona, Spain; Brown University School of Public Health, Providence, USA; European Observatory on Health Systems and Policies, London, UK; European Observatory on Health Systems and Policies, London, UK; Department of Health Services Research and Policy, London School of Hygiene and Tropical Medicine, London, UK; Health Systems Governance and Financing, World Health Organization, Geneva, Switzerland

## Abstract

**Background:**

The Covid-19 pandemic is an economic and a health crisis. Households reduced consumption expenditures as large-scale physical distancing measures, lower disposable incomes and fear of infection when engaging in many types of economic activity took hold. This, in turn, reduced domestic tax revenues at a time when governments were facing increased financial pressures to strengthen and sustain welfare states.

**Methods:**

We developed a simulation model, the Covid-19 Taxination Simulator, to estimate potential economic gains and tax revenues attributable to vaccine rollouts. We apply the model to 12 European Union countries which had low vaccination rates at the beginning of 2022.

**Results:**

The highest growth in aggregate personal consumption expenditure attributable to Covid-19 vaccines administered as of January 2022 is in Greece (10.8%), Slovenia (8.6%) and Czechia (8.6%), while the lowest is in Bulgaria (2.2%) and Slovakia (2.1%). If countries had vaccinated 85% of their adult population, the largest gains in consumption tax revenues would be expected in Romania (830 million Euros) and Poland (738 million Euros). Consumption tax revenues generated by meeting the 85% of the adult population target would, on their own, be large enough to fully cover the costs of expanding the vaccine rollout itself in Estonia, Latvia, Slovenia, Croatia, Czechia, Hungary and Greece.

**Conclusion:**

Covid-19 vaccination rollouts not only save lives and relieve pressures on health systems, they also support economic growth and generate additional tax revenues. These revenues can partially offset the costs of vaccines programmes themselves.

## Introduction

The Covid-19 pandemic has been a health and economic catastrophe. In the European Union (EU), real GDP contracted by 6.9% in 2020, with the largest declines in Spain (–10.8%), Greece (–9.0%), Italy (–8.9%) and Portugal (–8.4%). Household consumption expenditure is a main driver of these economic contractions. Declines in household consumption expenditure accounted for more than 100% of the decline in GDP in Q2 2020 in Latvia and Lithuania (124.4% and 181.9% of GDP growth, respectively), indicating that GDP would have fallen even further had it not been buoyed by growth in other non-consumption-related factors. More than half of the Q2 2020 GDP decline in Poland, Greece, Slovenia, Estonia and Romania was attributable to declining household consumption (see Supplementary figure S1). Lower consumer confidence has been attributed to a mix of large-scale physical distancing measures (e.g. lockdowns), lower disposable incomes and importantly, fear of risks of infection when engaging in many types of economic activity.^1^

For most countries, the way out of the Covid-19 economic crisis has depended on consumption expenditure returning to pre-pandemic levels; the key question is how? A working paper from the European Central Bank suggested that as of July 2020, without effective interventions, consumption could remain low in the long-term.^2^ Additionally, research suggests that traditional macroeconomic tools to stimulate consumer demand, such as stimulus payments or wage protection programmes, had limited effectiveness in supporting the economy during the pandemic.^3^

Vaccines are an essential component of a comprehensive strategy to achieve a return to pre-pandemic levels of consumption expenditure.^4^ Indeed, in many countries, vaccinations played a major role in enabling a re-opening of the economy and a return to more normal consumption patterns during 2021 as people began to feel safer coming into contact with others, leading to positive economic growth.^5^^,^^6^ According to Eurostat data, more than 80% of the return to GDP growth in Q2 2021 in Latvia and Lithuania was driven by positive growth in household consumption expenditure (84.5 and 83.1% of GDP growth, respectively) (see Supplementary figure S1). Hungary appears an exception, with just a quarter of GDP growth in Q2 2021 explained by changes in household consumption expenditure but this is because increased consumption was accompanied by extremely high growth in net exports, resulting in unusually high GDP growth.^7^ While the emergence of the Omicron variant may have reversed some economic progress in Europe at the end of 2021, the rapid rollout of boosters in some countries, with the accompanying increased immunity, played a crucial role to restore the situation to some extent in the subsequent months.

However, many countries were slow to vaccinate their populations, dampening their economic rebound. Moreover, boosters and, more likely, redesigned vaccines may be needed in the future,^8^ and without repeated vaccine rollouts, countries may find themselves returning to consumption patterns more in line with those seen in 2020 in future years, especially if new variants take hold. Some countries, such as the UK, have expressed concerns about fiscal pressures if they are to continue vaccine programmes in the long-term.^9^

In this paper, we estimate the potential personal consumption expenditure gains and subsequent tax revenues that may be achieved through Covid-19 vaccine rollouts. We achieve this by developing a simulation model of personal consumption expenditures, which we refer to as the Covid-19 Taxination Simulator. The simulator was commissioned through the ACT Accelerator to stimulate discussions between Ministries of Health and Ministries of Finance and ensure sufficient funding for COVID-19 vaccination roll outs.^10^ It has been used in low-income country settings to demonstrate the magnitude of return on investments of COVID-19 vaccination roll outs. Like other models used in the pandemic, the simulator should not be seen as a means to generate confident predictions but rather as a tool to explore plausible scenarios, in this case, the potential magnitude of economic and tax gains associated with different vaccine rollout strategies, which is otherwise very difficult in the absence of relevant historical data or individual-level consumption data linked with vaccination status.

As an illustration, we calculate estimates for 12 EU countries that had vaccinated fewer than 80% of their adult population as of the first week of January 2022: Bulgaria, Croatia, Czechia, Estonia, Greece, Hungary, Latvia, Lithuania, Poland, Romania, Slovakia and Slovenia and consider the potential annual consumption expenditure and tax effects under a range of scenarios. These countries provide an ideal setting for demonstrating the utility of the simulator because politicians have viewed the vaccine uptake achieved as sufficient to allow re-opening of the economy, even though others caution that there remain large numbers of people who remain unvaccinated. To our knowledge, there are no other studies that aim to investigate economic and tax implications of vaccine rollouts.

## Methods

The Covid-19 Taxination Simulator is a simulation model that estimates changes in personal consumption expenditure before and after Covid-19 vaccine rollouts to forecast potential economic gains and consumption tax revenues. The rationale for the modelling approach is that the economic crisis associated with the Covid-19 pandemic was initially driven by declines in consumption expenditure due to enforced restrictions or individual decisions to reduce risks of infection associated with many economic activities. Once a person is vaccinated, one might expect that person to return to pre-pandemic consumption behaviours as the reduced risk of experiencing severe disease following participation in economic activities increases consumer confidence. This consumption expenditure is not only a main driver of economic growth, but also increases consumption tax revenues.

For each country, we create a simulated population that mirrors that country’s population distribution by 5-year age group in 2020 according to Eurostat data. Each simulated person is assigned a baseline 2019 level of personal consumption expenditure according to their age group. Average personal consumption expenditures by age group are calculated from Household Budget Survey (HBS) data and derived for each simulated person probabilistically based on the inverse of the normal cumulative distribution for the mean and standard deviation of per person consumption expenditure among HBS survey respondents within their age group.

To estimate 2020 personal consumption expenditures for a simulated individual, we assume individuals restrict their spending to basic needs—in this case, food, housing and education—plus some additional non-basic needs consumption. This is consistent with research showing that households reduced all types of consumption expenditure during the pandemic, except for groceries.^11^ We do not include health expenditure as a basic need because of the disruptions to health care utilization during the pandemic.^12^ Average basic needs expenditures by age group are derived and assigned to individuals analogously to the approach used for average personal consumption expenditures by age group described above. Personal consumption expenditure levels for non-basic needs are calibrated so that, at a country level, the decline in simulated consumption expenditure between 2019 and 2020 equates to 50% of the observed reduction in personal consumption expenditure for each country according to Eurostat data. We choose 50% in the absence of data as a conservative estimate of the magnitude of the decline in personal consumption that can be influenced through vaccinations.

We then simulate vaccination rollouts. In our model, only individuals aged 15+ are eligible to be vaccinated. While we recognize that many countries have approved vaccines for those over age 5, we focus on adults only because children do not typically make their own spending decisions. We restrict the supply of vaccines in the model to match either actual vaccination rates as of January 2022 or aspirational rates—in this study, 85% of the adult population. Vaccines are randomly allocated among those who are eligible until pre-defined vaccination rates are achieved.

Vaccinated individuals return to their baseline 2019 consumption level in real terms. Those who accrued savings in the model—i.e. ‘wealthier’ individuals with high non-basic needs consumption expenditure at baseline—also spend a randomly generated share of their 2020 savings to reflect pent-up demand. Personal consumption is summed across the entire population and used to calculate aggregate annual growth attributable to a vaccination rollout. To calculate consumption tax revenues, we apply each country’s Standard VAT rates to aggregated consumption expenditures.

Some countries altered Standard VAT rates during the pandemic in an effort to incentivize households to spend, although research suggests VAT cuts aimed at restoring consumption levels are unlikely to have led to much additional consumption expenditure in Europe.^2^ To estimate potential tax losses resulting from VAT reductions, we simulated estimates of aggregate consumption tax revenues and per vaccinated person consumption tax revenues for Bulgaria, Czechia and Greece using both their Standard VAT and reduced Covid VAT rates. These countries were chosen due to the multi-sector inclusion of non-essential goods and services in their reduced Covid rates. Bulgaria adopted a 10% rate for hospitality, restaurants, cafes and some entertainment services.^13^ Czechia adopted a 10% rate for accommodation, sporting events, cultural activities and certain restaurant services. Greece adopted a 13% rate for cinema admissions, certain restaurant services and sporting events.^14^ We note that, in applying these reduced rates to all types of consumption expenditure, the model will underestimate potential tax revenues, since many types of consumption expenditures will continue to be taxed at the Standard rate. We also note that the lower rates were also only adopted temporarily by countries.

Finally, we also compare potential tax revenues to an estimate of vaccine rollout costs. Unfortunately, data on the costs of vaccine rollouts are not systematically available across countries. To estimate them, average per person vaccine costs within the EU were calculated using available figures for Moderna and Pfizer doses.^15^ AstraZeneca and Johnson & Johnson were not included due to the European Commission’s decision to terminate their contracts this year and focus on mRNA vaccines.^16^ This level was doubled to account for the two doses required for full vaccination. We also needed to account for logistics and other administrative costs. Estimations from the National Audit Office suggest the UK was to spend a total of £11.7bn on its vaccination programme, to buy and deploy 267 million doses, with £2.9bn reported to cover the costs of purchasing vaccines.^17^^,^^18^ Based on these figures, we estimated that the overall cost of a COVID-19 vaccine programme would be four times the cost of the vaccines, although this is likely to be an overestimate, given that salaries and related costs will likely be lower in the countries being studied.

To capture the degree of probabilistic uncertainty due to randomness in both individual consumption levels and vaccine uptake, we ran 100 iterations for each country’s vaccination rollout scenario. We use the median value from these iterations as our point estimates and report 95% uncertainty intervals.

The simulation tool was developed using Visual Basic. We report all growth rates in real terms (2019 prices) and tax revenue levels in 2019 Euros. Estimates reflect a counterfactual scenario of a year with no vaccine rollout (e.g. 2020). All country input parameters used for the simulation can be found in Supplementary table S1. No ethical approval was required.

## Results

**Figure 1:**
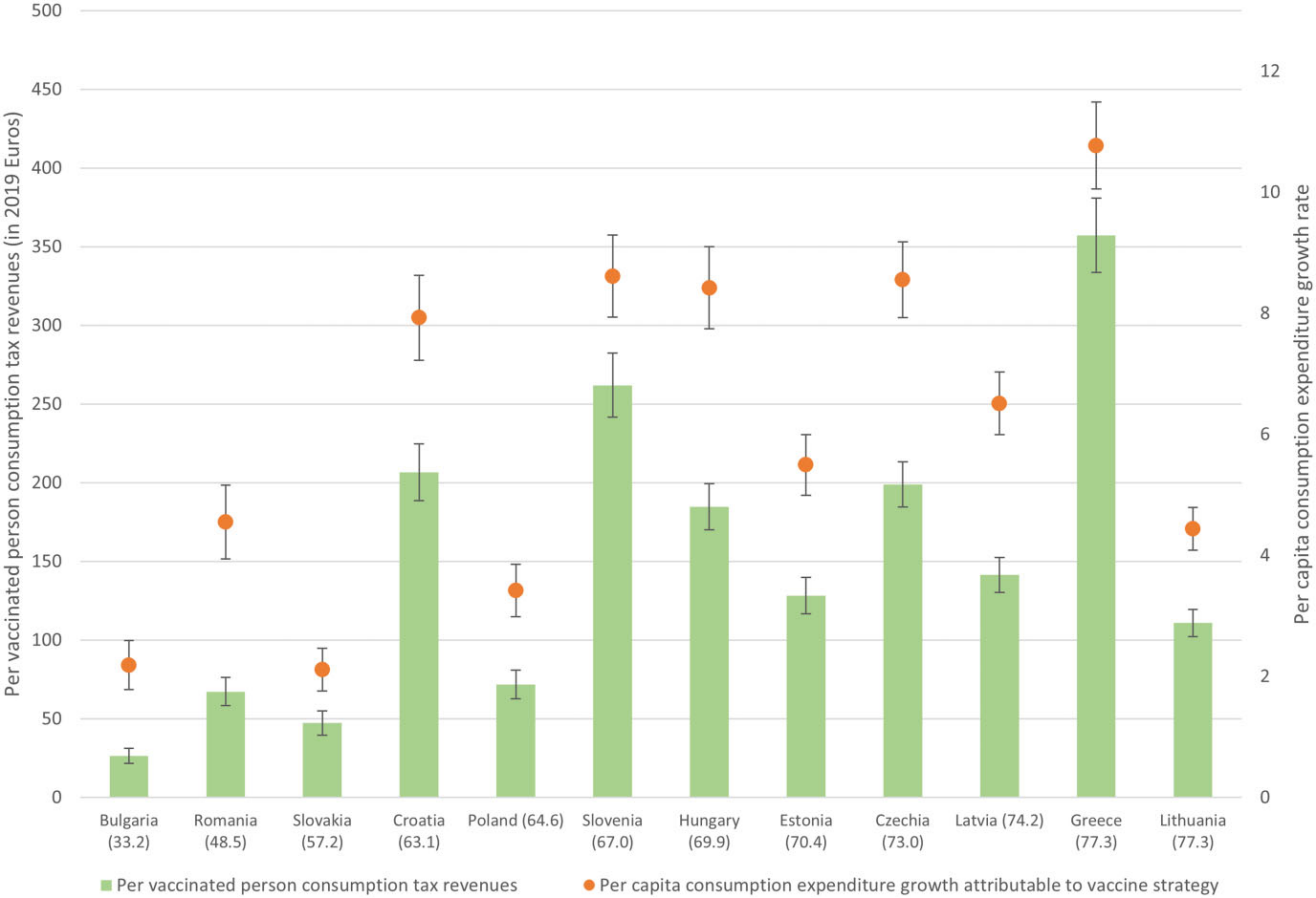
Consumption expenditure growth and tax revenues based on baseline (January 2022) vaccination rates Notes: Percent of adult population vaccinated as of January in parentheses.

Figure 1 contains estimates of potential consumption expenditure growth and tax revenues per vaccinated person for 2021 for the 12 countries based on actual vaccination rates as of January 2022. The highest estimated growth in aggregate consumption expenditure due to vaccine uptake is expected in Greece (10.8%), Czechia (8.6%) and Slovenia (8.6%), while the lowest is in Slovakia (2.1%) and Bulgaria (2.2%). Greece and Czechia are two of the most vaccinated countries in this study; however, Lithuania also had a similar share of its population vaccinated, but around half the potential consumption expenditure growth of either Greece or Hungary. This is because consumption in Lithuania fell less in 2020 than many other countries, while Greece had the largest year-over-year decline in household consumption in 2020 (–16.5%) among the 12 countries. Per person consumption tax revenues are estimated to be highest in Greece (357 Euros), well above the next highest country, Slovenia (262 Euros). The lowest per person consumption tax revenues attributable to vaccines are expected in Bulgaria (26 Euros), which had the lowest share of its population vaccinated as of January 2022.

**Figure 2:**
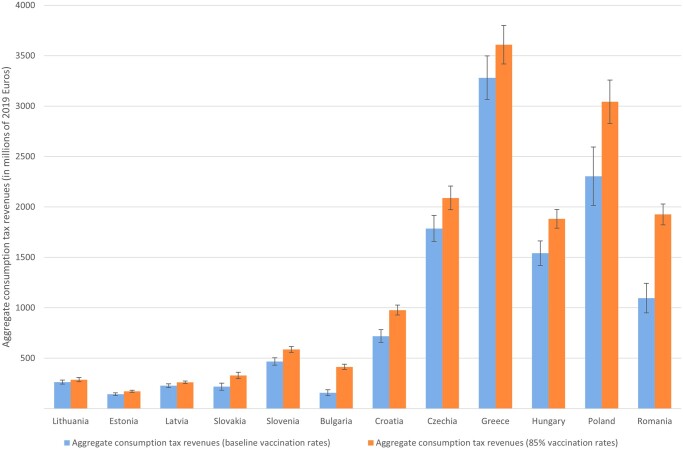
Aggregate consumption tax revenues attributable to baseline (January 2022) vaccination rates and target vaccination rates

Figure 2 presents estimates of aggregate consumption tax revenues given January 2022 vaccination rates compared with aggregate consumption tax revenues if countries were to meet a target of vaccinating 85% of their population over age 15. At both baseline vaccination rates and target vaccination rates, Greece has the highest potential level of tax revenues, 3.3 billion Euros and 3.6 billion Euros, respectively. To put this in context, the increase in tax revenues attributable to meeting the 85% vaccine target would be approximately 2.1% of 2019 VAT revenues in Greece (15.4 billion Euros).^19^ The greatest increases in tax revenues from meeting the target vaccination rate would be expected in Romania (830 million Euros) and Poland (738 million Euros). Romania had the second lowest vaccination rate in the EU at baseline (48.5%); however, Poland at baseline was higher at 64.6%. Compared with January 2022 vaccination rates, meeting the 85% vaccine target would be expected to generate 1.7% of Poland’s 2019 VAT tax revenues and 6.0% of Romania’s 2019 VAT revenues. The smallest increase in aggregate tax revenues would be expected in the Baltic States, owing to their small size.

**Figure 3:**
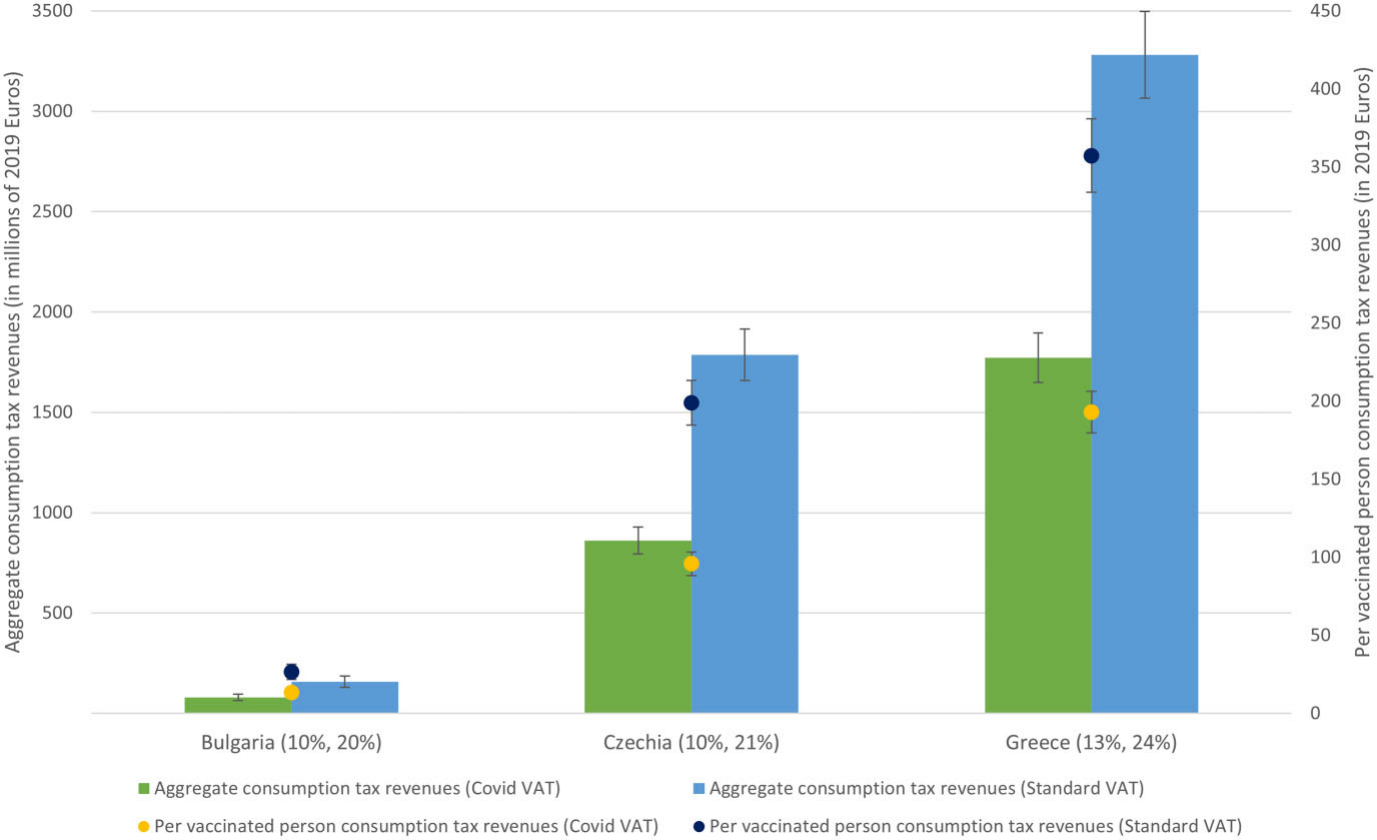
Aggregate consumption per person vaccinated tax revenues attributable to Covid VAT and Standard VAT at baseline vaccination rates Notes: Covid and Standard VAT rates in parentheses.

Figure 3 contains estimates of aggregate and per vaccinated person consumption tax revenues under Covid VAT and Standard VAT rates at baseline vaccination levels. Assuming negligible effects of reduced VAT on consumer demand, reducing VAT rates will have led to lower tax revenues as vaccines were rolled out. In Czechia, for example, 1.8 billion Euros could have been generated under Standard VAT rates at the level of vaccine uptake as of January 2022; however, at reduced VAT rates, this would fall to just 861 million Euros.

**Figure 4:**
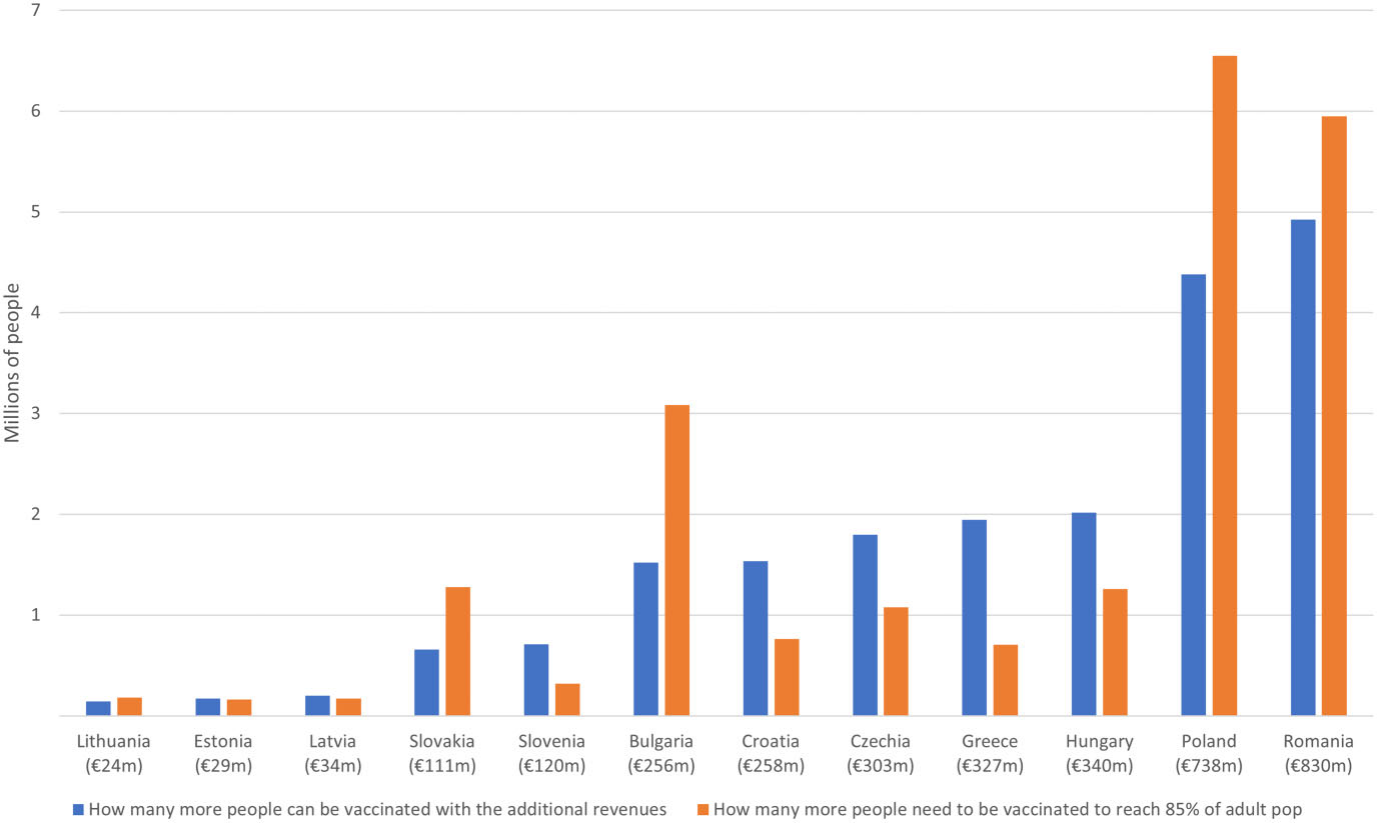
How many more people need to be vaccinated to reach 85% of the adult 15+ population and how many more people can be vaccinated using the additional revenues gained Notes: Covid and Standard VAT rates in parentheses.

Finally, we compare estimates of the number of people who need to be vaccinated to reach 85% of the age 15+ population targets with the number of people whose vaccinations could be financed through the potential tax revenues generated by the vaccine rollout expansion itself (figure 4). Our estimates suggest that the consumption tax revenues attributable to expanding vaccine rollouts to meet the 85% of the adult population target would fully cover the costs of the expansion in Estonia, Latvia, Slovenia, Croatia, Czechia, Greece and Hungary. In Lithuania, Slovakia, Bulgaria, Poland and Romania, the tax revenues generated through vaccinating more people partially offset the costs of expanding the vaccine rollout. For example, in Romania, approximately 83% of the cost of meeting the 85% target could be generated by the vaccine expansion itself.

## Discussion

The Covid-19 economic crisis has affected all countries in Europe and led to increased fiscal pressures as countries financed their pandemic response while maintaining essential public services. With growth in inflationary pressures and increases in the costs of borrowing,^20^ opportunities for external financing naturally decline and countries become more reliant on domestic sources of revenue. These revenues are needed both to finance regular government expenditures as well as to finance vaccine rollout expansions and boosters going forward, as well as to finance other Covid-19 related expenditures.

The methodological approach underpinning the Covid-19 Taxination Simulator reflects emerging evidence that vaccine rollouts contribute to a return to pre-pandemic consumer behaviours^21^^,^^22^; the results can provide useful insights for policymakers considering the costs and benefits of expanding vaccine rollouts. Policymakers have already expressed concerns about the costs of vaccine programmes, particularly with boosters or redesigned vaccines needed for the foreseeable future.^9^ While vaccinations are not without cost, we see that there are considerable economic and tax benefits attributable to widespread vaccine rollouts. Although the health risks of Covid-19 are particularly high for older people and people with pre-existing conditions, there are economic and fiscal gains—in addition to health—associated with more widespread rollouts.

We demonstrate that in all countries, the economic and tax gains attributable to vaccines either fully or partially offset the costs of vaccine rollouts. Whether countries choose to return these sums to the health system is a political choice. Health policymakers should be empowered by these findings to request extra funds from Ministries of Finance to support vaccination programmes; for example, a portion of vaccine-attributable tax revenues could usefully be allocated to support vaccine budgets in many countries.

The potential economic and tax gains vary by country and are not simply a function of increases in vaccination rates. This has various explanations, including differences in the size of the economy, the 2020 economic contraction, VAT levels and population demographics. For example, although we observe similar vaccination rates in Lithuania and Greece, Greece’s potential per capita consumption expenditure growth rate is more than twice that of Lithuania. This is to a considerable degree due to the much larger decline in consumption expenditure in Greece in 2020 and suggests that countries that managed the pandemic successfully will likely see smaller economic and tax gains of vaccine rollouts. At the same time, these countries will not be able to manage the pandemic long-term in the absence of vaccines, particularly if new variants that cause more severe illness take hold, and so, for these countries, using 2020 as a counterfactual scenario may not fully capture the value of vaccinations.

While this analysis is intended to be exploratory, it has several limitations. First, we assume that consumption expenditures of vaccinated individuals return to pre-pandemic levels. However, many consumption behaviours and preferences have changed during the pandemic, including increased online commerce, and it is possible that these types of changes in consumption behaviours will persist post-vaccination, which may affect both aggregate consumption levels and tax revenues. Similarly, in estimating potential consumption, we assume all consumption can be realized or, alternatively, that the supply of goods to be consumed is perfectly elastic. This may not be possible given large scale supply-chain disruptions^23^ and a lack of access to some goods due to lockdowns, with resultant inflationary pressure. This could suggest that we are overestimating the potential consumption effect in contexts where consumption expenditure is constrained for non-vaccine-related reasons. However, this will not materially affect the analysis, both because we are studying countries for whom a large share of the population is already vaccinated and which were broadly re-opened for business, and because we are interested in the potential tax revenues once 85% of the adult population is vaccinated, by which time we would expect many aspects of the economy to have re-opened anyways, supported by continued safeguards such as improved ventilation, mask wearing in certain settings and effective testing regimes.

Perhaps the greatest limitation, and one that makes our estimate highly conservative, is that it only captures individual decisions by adults based on their vaccination status. Yet the benefit of being vaccinated is not confined to the individual. Even if the effect of vaccination on onward transmission by those infected with new variants is sub-optimal, it will likely reduce their risk of becoming infected and infecting others. In this way, a high level of vaccination may give many more people confidence to engage in economic activities. Relatedly, as this analysis is illustrative, we have held all else equal although clearly it is not and, in reality, confidence will be influenced by other policies that themselves impact on the amount of infection in the population.

Additionally, we assume all consumption is taxed at each country’s Standard VAT rates, which may not reflect reality since some types of consumption are untaxed, and some types of consumption are taxed at reduced rates. Future work should separate out different types of consumption and tax them accordingly; however, with limited rationale for adjustments to different forms of consumption expenditure, we opted to treat all consumption the same. Similarly, we applied the Covid-reduced VAT rates (figure 3) to all sectors, which is again an oversimplification where sectoral rates, for example for dining out, were reduced only temporarily.

A further caveat is that in the comparison of tax revenues with costs, we make no explicit estimate of costs of wastage, nor do we vary costs of administering doses across countries.

Finally, we have focused exclusively on consumption effects. Future analysis should consider broader economic and tax effects, particularly in countries where exports or tourism are the main economic drivers.

Covid-19 vaccination rollouts matter, not only to save lives and relieve pressures on health systems, but also to support economic growth. While economic growth is inadequate on its own as a singular measure of societal well-being, to inform decisions in a context of finite resources, it is important to take note of both the costs as well as the range of benefits associated with policy interventions. Based on this analysis, vaccines should be seen as a form of economic stimulus, not only as a cost, as is too often the case with health care expenditures.^24^ Even if boosters were to be needed indefinitely, financing for vaccines should not substitute for spending on other types of health services. Additional financing is needed, and as shown through this analysis, the resources to pay for vaccine rollouts are adequately generated as a result of the vaccines themselves.

## Supplementary Material

ckad023_Supplementary_Data
