# Supplementary material for: Consumption and tax gains attributable to Covid-19 vaccinations in 12 EU countries with low vaccination rates
Source: Eur J Public Health. 2023 Feb 13;33(2):228–34. doi: 10.1093/eurpub/ckad023 (PMC10066489; doi:10.1093/eurpub/ckad023)
Supplement: ckad023_Supplementary_Data [file ckad023_supplementary_data.docx]

**Annex**

**Figure 1. Quarter 2 GDP growth rates and GDP growth attributable to changes in household consumption**

Note: Percentage point change compared to same period in previous year; seasonally and calendar adjusted. Source: Eurostat

**Table 1. Country input parameters used for simulation**

|  |  | Bulgaria | Romania | Slovakia | Croatia | Poland | Slovenia | Hungary | Estonia | Czechia | Latvia | Greece | Lithuania |
| --- | --- | --- | --- | --- | --- | --- | --- | --- | --- | --- | --- | --- | --- |
| Population by age groups | ***0-4*** | 320507 | 1002521 | 294128 | 182408 | 1921497 | 100787 | 468237 | 71235 | 568823 | 103060 | 461955 | 145590 |
|  | ***5-9*** | 339533 | 966274 | 290061 | 196674 | 1942294 | 110259 | 456162 | 72778 | 563395 | 99627 | 511958 | 144512 |
|  | ***10-14*** | 342218 | 1062583 | 279531 | 202389 | 1998280 | 104756 | 496937 | 74689 | 577984 | 102501 | 553606 | 132642 |
|  | ***15-19*** | 312977 | 1025575 | 262751 | 198576 | 1786579 | 93729 | 487664 | 62917 | 478030 | 89522 | 549705 | 130926 |
|  | ***20-24*** | 301636 | 1001902 | 292244 | 238386 | 2018851 | 103089 | 541142 | 63051 | 483032 | 83996 | 559606 | 154360 |
|  | ***25-29*** | 390614 | 1054739 | 367835 | 238457 | 2508023 | 113876 | 630891 | 81682 | 651892 | 115546 | 564676 | 185376 |
|  | ***30-34*** | 476452 | 1370847 | 411370 | 255340 | 2870306 | 136265 | 619934 | 101265 | 722127 | 137794 | 596438 | 187446 |
|  | ***35-39*** | 479936 | 1323981 | 436101 | 280875 | 3139152 | 152537 | 651992 | 95087 | 762998 | 130379 | 736795 | 171793 |
|  | ***40-44*** | 532837 | 1536588 | 455315 | 277900 | 2962472 | 159721 | 837893 | 91061 | 923446 | 124087 | 799789 | 174252 |
|  | ***45-49*** | 522513 | 1483204 | 398549 | 266906 | 2555771 | 148966 | 749855 | 91734 | 839278 | 131906 | 799716 | 195470 |
|  | ***50-54*** | 479580 | 1479299 | 346980 | 280613 | 2233905 | 152755 | 663102 | 83813 | 686236 | 127715 | 797821 | 201308 |
|  | ***55-59*** | 473775 | 1062251 | 357920 | 290286 | 2372474 | 150370 | 572104 | 87640 | 653501 | 138282 | 717651 | 217731 |
|  | ***60-64*** | 474816 | 1297311 | 359913 | 295571 | 2731788 | 144747 | 651379 | 85736 | 651567 | 131847 | 682649 | 196708 |
|  | ***65-69*** | 463819 | 1201894 | 329405 | 267320 | 2447824 | 134197 | 643679 | 77501 | 678927 | 109804 | 609002 | 154540 |
|  | ***70-74*** | 418088 | 894266 | 231241 | 203186 | 1748149 | 96407 | 482487 | 62709 | 602974 | 90370 | 568279 | 126480 |
|  | ***75-79*** | 283876 | 636767 | 159849 | 160970 | 1035014 | 79233 | 377278 | 48633 | 408629 | 80187 | 435679 | 110410 |
|  | ***80-84*** | 192555 | 525103 | 101104 | 129098 | 876987 | 60031 | 239194 | 42511 | 234495 | 64080 | 395824 | 90739 |
|  | ***85+*** | 145750 | 403733 | 83576 | 93210 | 808772 | 54136 | 199596 | 34934 | 206605 | 46972 | 377416 | 73807 |
|  | ***Total 15+*** | 5949224 | 16297460 | 4594153 | 3476694 | 32096067 | 1780059 | 8348190 | 1110274 | 8983737 | 1602487 | 9191046 | 2371346 |
| VAT rate (%) |  | 20 | 19 | 20 | 25 | 23 | 22 | 27 | 20 | 21 | 21 | 24 | 21 |
| Covid VAT rate (%) |  | 10 |  |  |  |  |  |  |  | 10 |  | 13 |  |
| 2019 personal consumption expenditure (€) |  | 5416.24 | 6992.68 | 9681.09 | 9709.28 | 8012.76 | 12781.58 | 7512.87 | 10281.70 | 10067.85 | 9229.79 | 12977.05 | 10503.96 |
| 2020 consumption growth* |  | -4.850 | -6.36 | -2.62 | -8.26 | -3.98 | -7.80 | -7.60 | -5.38 | -7.87 | -5.73 | -9.03 | -3.85 |
| Adult vaccine uptake 7/1/22 (%) |  | 33.2 | 48.5 | 57.2 | 63.1 | 64.6 | 67 | 69.9 | 70.4 | 73 | 74.2 | 77.3 | 77.3 |

Notes: *calculated as the difference between the 2019-2020 PCE growth and the 2014-2019 average PCE growth, multiplied by 0.5. Sources: Eurostat, ECDC, OECD, Avalara.
